# Comparative Analysis of Retinal Organotypic Cultures and In Vivo Axotomized Retinas

**DOI:** 10.3390/ijms24043481

**Published:** 2023-02-09

**Authors:** María José González-Riquelme, Fernando Lucas-Ruiz, Caridad Galindo-Romero, Raquel Boia, António Francisco Ambrósio, Manuel Vidal-Sanz, Ana Raquel Santiago, Marta Agudo-Barriuso

**Affiliations:** 1 Experimental Ophthalmology Group, Instituto Murciano de Investigación Biosanitaria Virgen de la Arrixaca (IMIB-Arrixaca) & Universidad de Murcia, 30120 Murcia, Spain; 2 University of Coimbra, Coimbra Institute for Clinical and Biomedical Research (iCBR), Faculty of Medicine, 3000-548 Coimbra, Portugal; 3 University of Coimbra, Centre for Innovative Biomedicine and Biotechnology (CIBB), 3000-075 Coimbra, Portugal; 4 Clinical Academic Centre of Coimbra (CACC), 3000-075 Coimbra, Portugal; 5 Association for Innovation and Biomedical Research on Light and Image, 3000-548 Coimbra, Portugal; 6 University of Coimbra, Institute of Immunology, Faculty of Medicine, 3000-548 Coimbra, Portugal

**Keywords:** microglia, Müller cells, retinal ganglion cells, astrocytes, axotomy, in vivo, in vitro

## Abstract

Retinal organotypic cultures (ROCs) are used as an in vivo surrogate to study retinal ganglion cell (RGC) loss and neuroprotection. In vivo, the gold standard to study RGC degeneration and neuroprotection is optic nerve lesion. We propose here to compare the course of RGC death and glial activation between both models. The left optic nerve of C57BL/6 male mice was crushed, and retinas analyzed from 1 to 9 days after the injury. ROCs were analyzed at the same time points. As a control, intact retinas were used. Retinas were studied anatomically to assess RGC survival, microglial, and macroglial activation. Macroglial and microglial cells showed different morphological activation between models and were activated earlier in ROCs. Furthermore, microglial cell density in the ganglion cell layer was always lower in ROCs than in vivo. RGC loss after axotomy and in vitro followed the same trend up to 5 days. Thereafter, there was an abrupt decrease in viable RGCs in ROCs. However, RGC somas were still immuno-identified by several molecular markers. ROCs are useful for proof-of-concept studies on neuroprotection, but long-term experiments should be carried out in vivo. Importantly, the differential glial activation observed between models and the concomitant death of photoreceptors that occurs in vitro may alter the efficacy of RGC neuroprotective therapies when tested in in vivo models of optic nerve injury.

## 1. Introduction

The retina has many advantages over other areas of the central nervous system (CNS) to study the intrinsic mechanisms of neurodegeneration and neuroprotection. It is accessible with relative ease, its anatomy is very well-known, and it can be treated locally with intravitreal or subretinal injections, or systemically if the drug crosses the blood-retinal barrier [1,2,3,4,5]. Anatomical and functional changes can be followed up longitudinally in vivo [6,7] and, in post mortem samples, molecular and further anatomical studies can be performed [3,4,8,9]. Moreover, there are many standardized retinal models of injury and disease [6,10,11,12,13]. 

Nowadays, one of the challenges in animal experimentation is to apply the 3Rs principle for responsible and quality research. Working in vivo implies the use of animals, in general rodents. As a rule, only one retina is injured while the contralateral retina one has been traditionally used as a control. However, in recent years, it has been shown that there is a contralateral detrimental response in the contralateral retina to the lesions [2,3,14,15,16,17]. Thus, ideally controls must be retinas from intact animals, increasing the number of animals per study.

The advantage of using in vivo models is that we have the entire system where all cell interactions and systemic effects are in play. Alternative ex vivo models such as organoids or organotypic cultures lack the complete physiological crosstalk but have other benefits. In addition to providing better compliance with the 3Rs, ex vivo models allow working with human samples. Organoids can be derived from human cells, and post mortem retinas from donors can be cultured organotypically. 

Retinal organoids are currently intensively used in research as they allow studying human retinal development, therapeutic medicines, and advanced diagnosis by creating organoids bearing human mutations. They have, however, some drawbacks for studying neurodegenerative processes. Firstly, the culture process is very laborious, and a large scale of retinal organoids is very expensive and time-consuming [18]. Secondly, the ganglion cell layer (GCL) gradually degenerates, probably because of a lack of blood supply and synaptic connectivity with the retinorecipient targets. Thirdly, the retinal pigment epithelium (RPE) and cells of mesoderm origin (blood vessels and microglial cells), which are essential for maintaining retinal homeostasis, are absent in these organoids [19].

The other widely used ex vivo model is the retinal organotypic cultures (ROCs) that consist of culturing flat dissected retinas [20]. This method reduces the number of animals since four biological samples can be obtained from each donor by halving the retinas and culturing each half in a separate well. Importantly, human retinas from donors can be cultured. Contrary to retinal organoids, ROCs conserve the retinal blood vessels (but lose the blood perfusion) and microglial cells. Once the retina is removed from the eye, retinal ganglion cells (RGCs) suffer an axotomy and photoreceptors are separated from the RPE, causing retinal detachment. In addition, the retina is separated from the choroid and the vitreous is no longer bathing the inner retina. Regardless of these other elements, ROCs are mostly used as a model of RGC degeneration and to test new therapies [21,22,23] because treatments are very well controlled in vitro. 

One widely used in vivo model of RGC axotomy is crushing the optic nerve (optic nerve crush, ONC) [2,3,8,9,13]. This injury causes interruption of the axonal flow, anterograde and retrograde degeneration, and the death of RGC that follows the same course as after complete optic nerve transection [8,9]. Optic nerve transection involves opening the meninges surrounding the optic nerve to avoid severing the retinal artery. The advantage of ONC is that the meninges do not have to be opened, because the retina re-perfuses naturally after the crush [2,8,9,10].

How well do ROCs translate to in vivo models of RGC degeneration? To answer this question, we compared the course of RGC death and glial cell activation between an ONC model [8] and ROCs using standard methods for cell immunodetection and quantification in retinal neurodegeneration and neuroprotection analyses.

## 2. Results

### 2.1. Course of RGC Death: ROCs vs. ONC

**Figure 1:**
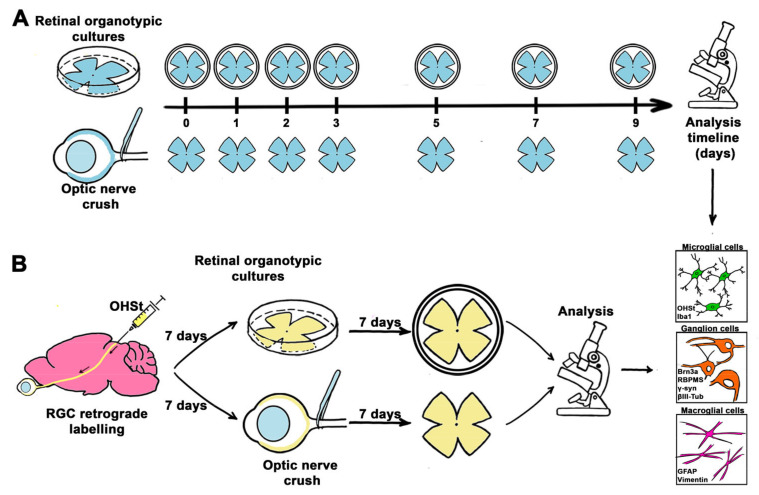
Experimental design. (**A**) Comparative study of RGCs and glial cells between ROCs and ONC. (**B**) Assessment of phagocytic microglial cells after ONC and in ROCs.

First, we quantified RGCs in intact retinas processed for ONC model and the in vitro study, as they were handled differently (Figure 1). Control intact retinas for ONC analysis were obtained from animals perfused transcardially, and in vitro retinas were fresh dissected from animals using the explant dissection technique but without placing them on the inserts (see materials and methods). Both samples were processed immediately for immunodetection. We immunodetected RGCs with the gold standard markers, Brn3a and Rbpms [10,12,24,25,26].

**Figure 2:**
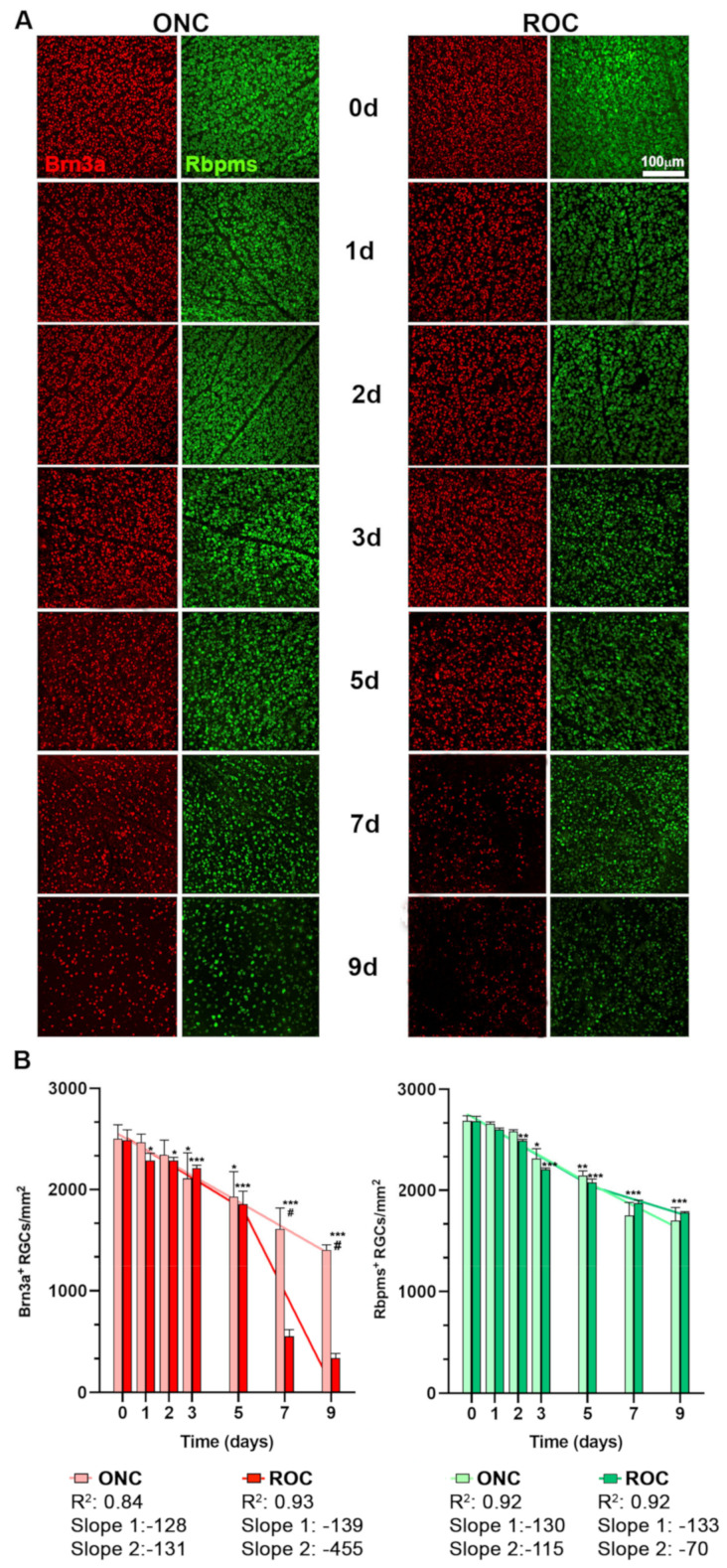
Course of retinal ganglion cell death in ROCs and after ONC. (**A**) Representative magnifications from intact retinas (day 0) and retinas analyzed from 1 to 9 days after ROC and ONC showing Brn3a (red) and Rbpms (green) positive RGCs. (**B**) X- and Y-graphs (time versus cells) showing the mean ± SD density (cells/mm^2^) of Brn3a + RGCs (left) and Rbpms + RGCs (right) in both groups. Time 0 represents data from control retinas. The linear regression was calculated in two phases (X0 at day 5). Slope 1 does not differ between models or markers. Slope 2 is significantly steeper in ROCs than after ONC for Brn3a, and after ONC than in ROCs for Rbpms (*p* < 0.0001). * Intact vs. experimental (* *p* < 0.05, ** *p* < 0.01, *** *p* < 0.001, Kruskal–Wallis test, Dunn’s multiple comparisons test); # ONC vs. ROC (# *p* < 0.001, Mann–Whitney test). *n* = 4–6 retinas/group/time point.

As shown in Figure 2, the dissection and fixation techniques did not affect RGC immunodetection. Therefore, although for each analysis we used the appropriate controls (i.e., perfused for ONC; freshly dissected for ROCs), we will call these intact retinas hereafter.

Of note, the mean density of Rbpms+RGCs is slightly higher than that of Brn3a+RGCs. Brn3a is only expressed by vision-forming RGCs while Rbpms are also expressed by intrinsically photosensitive RGCs (ipRGCs). In mice, ipRGCs represent 7.5% of all RGCs [26,27].

RGC death after ONC followed the course previously described by our laboratory [2,4,10,13], reaching statistical significance with both markers at 3 days after the injury. In ROCs, the loss of RGCs was significant at day 1 and day 2, when we immunodetect Brn3a or Rbpms antibody, respectively. (Figure 2A,B). 

At day 7 in vitro, the density of RGCs immunodetected with Brn3a dropped to a third of the density found after ONC. Surprisingly, this sharp decrease was not observed for Rbpms immunodetection. 

To better compare the kinetics of RGC death between both models and markers, we performed a two-segment regression analyses: from day 0 (intact values) to day 5, and from day 5 to day 9 (Figure 2B). The course of RGC loss after ONC was the same for both markers (difference between slopes *p* = 0.931). When comparing models and markers, regression analyses showed that RGC loss did not differ between models and markers until day 5 (slope -calculated loss of cell density/day- for Brn3a −128 (ONC) and −139 (ROCs); for Rbpms −130 (ONC) and −139 (ROCs); *p* > 0.05). Thus, during this time window (0–5 days), RGC loss follows the same kinetics in both models and with both markers. 

From day 5 to 7, there were differences between models and markers. When RGCs were identified with Brn3a, the course of death was significantly quicker in ROCs than after ONC (slopes −131 (ONC) and −455 (ROCs); *p* < 0.0001) and when RGCs were identified with Rbpms occurred the opposite: RGC loss was significantly quicker after ONC (slope −115) than in ROCs (slope −70; *p* = 0.048).

Then we calculated the ratio of Rbpms:Brn3a. In accordance with the regression analyses, this ratio ranged from 1.1 to 1.2 from baseline (intact) to day 9 after ONC and from baseline to day 5 in ROCs. However, at days 7 and 9 in ROCs, there were, respectively, 3.5 and 5.2 more Rbpms + RGCs than Brn3a + RGCs.

Brn3a is a transcription factor that is downregulated when the RGC is committed to death [4]. In other words, Brn3a is a viability marker, while Rbpms expression is somatic, and its downregulation has not yet been linked to RGC viability [26]. 

**Figure 3:**
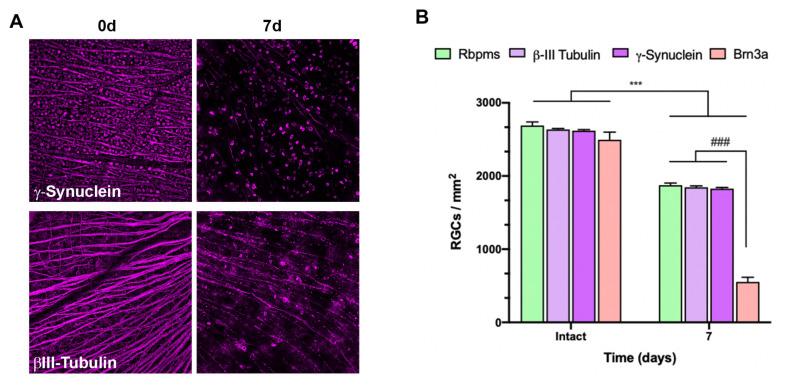
Structural RGC markers are immunodetected in ROCs after RGC death. (**A**) Representative magnifications from intact retinas and ROCs at day 7 showing γ-synuclein and β-III tubulin immunodetection (**B**) Bar graphs showing the mean ± SD density (cells/mm^2^) of RGCs immunodetected with each marker in intact retinas, and in ROCs after 7 days (images for Rbpms and Brn3a are shown in Figure 2). Intact vs. 7d (*** *p* < 0.001, Mann–Whitney test); Significant difference between markers at 7 days (### *p* < 0.001, Kruskal–Wallis, Dunn’s multiple comparisons test). *n* = 4–6 retinas/group.

To ascertain whether other structural proteins were still present in ROCs when RGCs had lost their viability, we analyzed the expression of β-III tubulin and γ-synuclein, both cytoskeletal proteins and RGC markers, both in intact retinas and in 7-day ROCs [28,29]. As seen in Figure 3, the density of RGCs labeled with these two markers did not differ from that found for Rbpms, which means that structural but not viability proteins were still present in the tissue.

### 2.2. Microglial Behavior in ROCs

Next, we assessed microglial cells only in the GCL of intact control retinas, ROCs, and after ONC, because microglia are important players in clearing dead RGCs from the tissue. 

**Figure 4:**
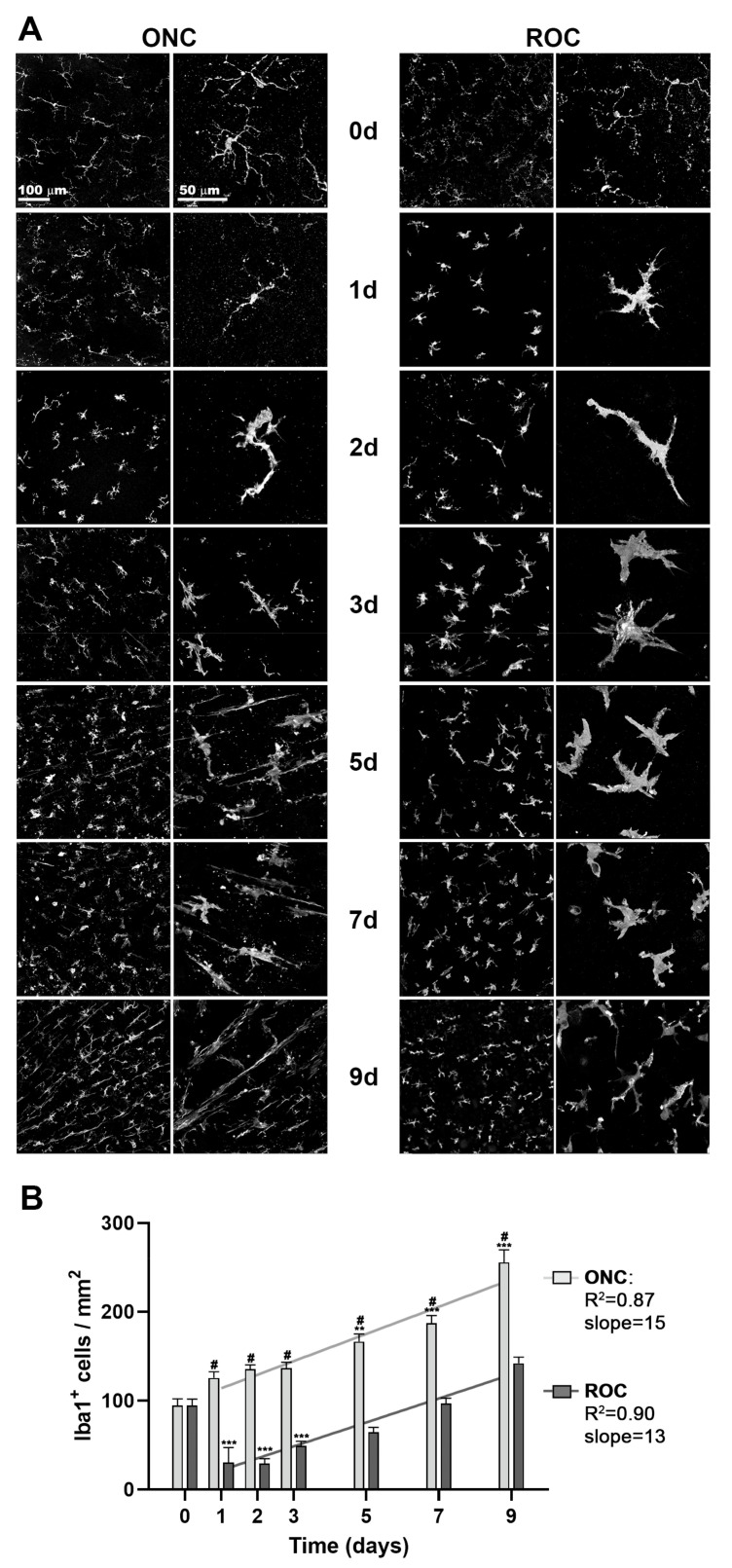
ROCs have a lower density of microglial cells in the GCL than axotomized retinas. (**A**) Representative magnifications from intact retinas (day 0) and retinas analyzed from 1 to 9 days after ROC and ONC showing Iba1+ microglial cells. (**B**) X- and Y-graphs (time versus cells) showing the mean ± SD density (cells/mm^2^) of Iba1+ cells in both groups. Time 0 represents data from intact retinas. After a significant decrease in Iba1 + cells between day 0 and 1 in ROCs, data adjust to a linear regression from day 1 onwards. The slope between both models is significantly steeper after ONC than in ROCs (*p* = 0.048). Experimental vs. intact (** *p* < 0.01, *** *p* < 0.001, Kruskal–Wallis test, Dunn’s multiple comparisons test); # ONC vs. ROC (# *p* < 0.001, Mann–Whitney test). *n* = 4–6 retinas/group/time point.

In ONC retinas, microglial cells showed early morphological changes associated with reactivity such as rounded somas and shorter and fewer branches than in their resting state (Figure 4A). From day 5 onwards, many microglial cells had an elongated morphology with two main processes and were found along the RGC axons, radiating from the optic nerve head, as already reported [30,31]. This topographical pattern was not observed in ROCs.

In ROCs, the microglial cells also showed early morphological changes, but their morphology differed from ONC retinas with somas that were larger and branches that were thicker. This engrossed morphology was observed at all time points and was especially clear at 3 days in vitro (Figure 4A). 

After ONC, the density of Iba1+ cells in the GCL increased linearly from day 1, as previously described [2,30]. In ROCs, there was a significant decrease in Iba1+ cells at day 1 and thereafter their density increased linearly (Figure 4B), but this density was at all time points significantly lower than in ONC retinas. 

In both models, the density of microglial cells increased with time. However, the increase of microglial cells was significantly higher after ONC than in ROCs (slopes of the lineal regresion: 13 for ROCs, and 15 for ONC; *p* = 0.048).

Microglial changes in morphology in ROCs, together with their diminished number, as well as the fact that RGCs structural markers were immunodetected when the RGC had already died, led us to hypothesize that in vitro microglial cells phagocytose fewer RGCs than after ONC. 

To test this hypothesis, first we traced RGCs with OHSt placed onto the superior colliculi and, once traced, we performed ONC or ROCs. Seven days later, we quantified Iba1 + OHSt+ microglial cells in all samples. OHSt is a fluorescent retrograde tracer that is not degraded by microglial cells upon phagocytosis of a traced-RGC and thus accumulates into their phagolysosomes [2]. 

**Figure 5:**
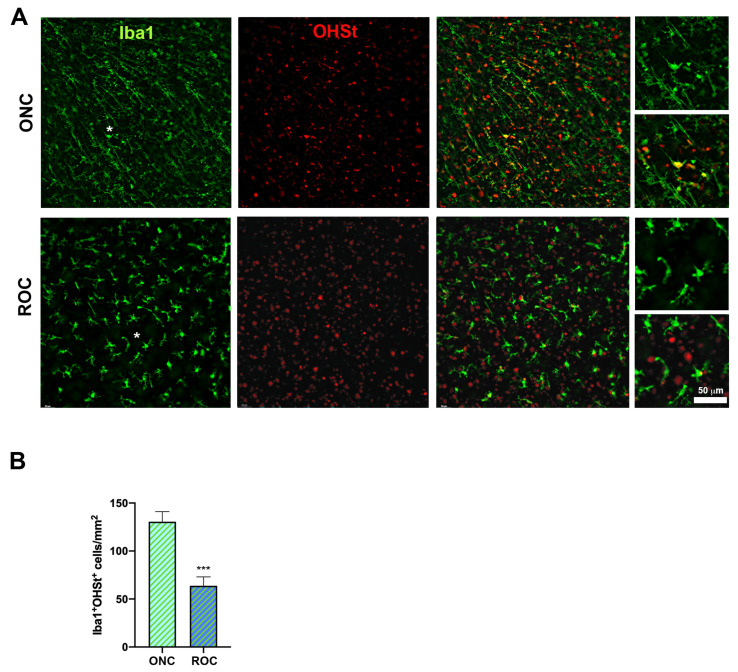
ROCs have a lower density of phagocytic microglial cells in the GCL than axotomized retinas. (**A**) Representative magnifications from traced retinas 7 days after ONC and ROC showing traced cells (OHSt, red) and Iba1 + microglial cells (green). Iba1 + OHSt+ cells are phagocytic microglial cells. Asterisks in the Iba1 panels mark the area from which the magnifications on the right were taken. (**B**) Bar graphs showing the mean ± SD density (cells/mm^2^) of phagocytic microglial cells in ROCs and after ONC. *ONC vs. ROCs (*** *p* < 0.001, Mann–Whitney test). *n* = 4–6 retinas/group.

Thus, co-localization of OHSt and Iba1 means that a microglial cell engulfed at least one traced-RGC [2,31]. As shown in Figure 5, there was a significantly higher density of Iba1 + OHSt+ microglial cells after ONC than in ROCs. To assess whether this was related to a lower number of microglial cells or to a decreased phagocytic activity, we calculated the ratio of the microglial cells: phagocytic microglial cells (Iba1+: Iba1 + OHSt+) at day 7. The ratio was similar in both models (1.5 in ROCs and 1.4 after ONC).

Thus, our data show that RGCs in ROCs are cleared off slower than after ONC because there are fewer microglial cells in the GCL.

### 2.3. Macroglial Activation

**Figure 6:**
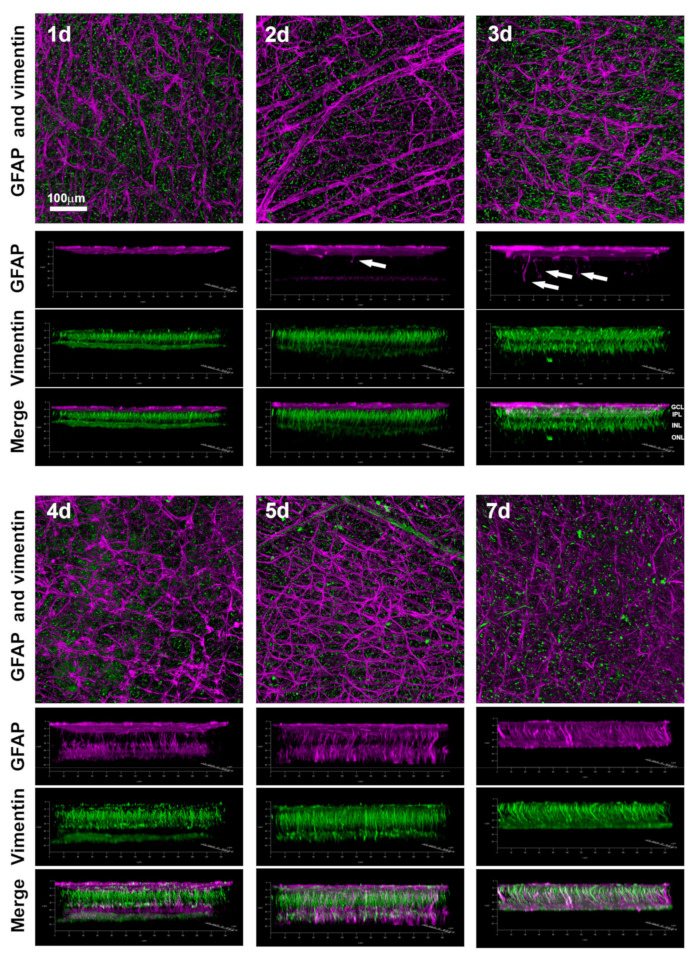
Early induction of macrogliosis in ROCs. Representative confocal microphotographs focused on the ganglion cell layer of ROCs immunodetected against GFAP (purple) and vimentin (green). Below each image, the 3D representations of the full thickness of each ROC are shown. Arrows point to reactive Müller cells. GCL: ganglion cell layer. IPL: inner plexiform layer. INL: inner nuclear layer. ONL: outer nuclear layer. *n* = 4–6 retinas/group/time point.

Finally, we analyzed Müller cell and astrocyte gliosis in ROCs over time. ROCs were double immunodetected for GFAP (astrocytes and activated Müller cells marker) and vimentin (Müller cells marker), both intermediate filaments that are upregulated in gliosis [32]. Astrocytes were hypertrophied from day 2 with thicker processes and stronger GFAP signal than at day 1, and by day 7 their coverage of the GCL decreased (Figure 6).

Müller cell activation was analyzed in the 3D confocal reconstructions. At days 2 and 3 after culture, some Müller cells were already hypertrophied, and their GFAP expression was observed along their somas and stem processes crossing the retina (Figure 6, arrows). From day 4 onwards, Müller cell activation was observed in the inner and outer retina with the GFAP signal spanning all retinal layers. In the 3D reconstructions of the confocal images, it was observed that at day 7 in vitro, when the number of viable RGCs dropped dramatically, ROCs had thinned considerably. 

We have recently published the course of macroglial activation after ONC [30]. Thus, to save animals we did not repeat those experiments here. Axotomized retinas showed an increase in GFAP and vimentin expression from days 5 to 9 in the GCL, indicating a hypertrophy of astrocytes and Müller cells’ endfeet. However, Müller cell gliosis did not extend to their somas and stem processes. Finally, after ONC there was not a visible retinal thinning up to 45 days after the lesion.

## 3. Discussion

Here we show, for the first time, a comprehensive anatomical comparison between ROCs and axotomized retinas. In addition, this is the first long-term study of neurodegeneration in ROCs. ONC and ROCs are used to assess RGC degeneration and to test neuroprotective therapies because, in both models RGCs are axotomized. However, they differ on important points that explain the differences observed in this work. While in vivo, the retina undergoes one injury, optic nerve axotomy; in vitro, there are other factors such as a disconnection from the body, i.e., loss of systemic crosstalk, retinal detachment, and lack of blood perfusion and vitreous support.

In the literature, the RGC fate in ROCs has been followed up to 7 days using the RGC markers that also label displaced amacrine cells [33] or are structural, such as β-III tubulin [26,34]. Pattamatta et al. used Rbpms and βIII-tubulin. Their results about Rbpms conflict with ours as they did not observe Rbpms staining further than 1 day in vitro. This difference could be due to the fixation and immunodetection protocols that differ between their work and ours. Our fixation was performed overnight at 4°C without any agitation so as not to damage the tissue. Additionally, ROCs were washed with a higher percentage of Triton^®^ X-100 for further membrane permeabilization. Thus, antibodies can more easily enter the cell, as Brn3a and Rbpms are nuclear and cytosolic antigens, respectively.

Regarding RGC survival in vitro and after ONC, in ROCs there is a massive wave of RGC death between days 5 and 7 that does not follow the course observed in axotomized retinas with Brn3a [2,3,8,9,13,25]. However, in 7 days ROCs, Rbpms and structural RGC markers are still present and thus immunodetected. This discrepancy between the RGC markers may be due to the fact that Brn3a is a marker of RGC viability [4,26], whereas, to date, Rbpms, β-III tubulin, and γ-synuclein have not been demonstrated to be viability markers.

ROC is a rather aggressive model and causes a quick and severe degeneration of the inner and outer retina, as seen here and elsewhere [35,36], while in vivo the environment is more stable for neurons [37,38]. 

RGC death after axotomy is quick and homogeneous thorough the retina, with a lineal kinetic in two phases, a quick one from 1 to 7 and 9 days, in mice and rats. respectively, and a slow one thereafter [2,3,8,9,13,39,40]. During the quick phase of RGC death, half of the original population is lost at 5 days in mice (7 in rats). We show here that up to 5 days, the loss of RGCs with both markers follows the same course and kinetics in ROCs and after ONC. Therefore, until day 5, there is a therapeutic window that is valid to study RGC degeneration or to test neuroprotective therapies. Long-term experiments, however, should be carried out in vivo.

In vivo, axotomy-induced RGC death exceeds the phagocytic capacity of resident microglial cells. Thus, to meet the requirements for resolving the damage, microglial cells migrate from the plexiform layers to the GCL [2,31,41] or from the optic nerve or by recruitment of systemic macrophages and myeloid cell infiltration [42,43]. In models of photoreceptor degeneration, which also cause a quick and extensive neuronal death, the microglial cell number increases by proliferation and there is also entry of macrophages from the choroid [44]. 

In ROCs, both RGCs and photoreceptors die [45] and resident microglial cells are mobilized to the inner and outer retina. Concomitant degeneration of RGCs and photoreceptors explains why the density of microglial cells in the GCL decreases at day 1 compared to intact retinas, since microglial cells migrate to the photoreceptor layer to phagocytose the dead photoreceptors. In ROCs, there is no microglial recruitment or turnover [46] explaining why even though microglial density in the GCL increases with time, it is at all time points lower and slower than after ONC. In other words, in ROCs, the number of resident microglial cells is not enough to phagocytose all dead RGCs at the same speed as in vivo. 

Therefore, and in accordance with our data, the phagocytosis of RGCs is diminished in ROCs because there are not enough microglial cells in the GCL. Thus, the debris of RGC somas is still present in the tissue and structural but not viability markers are immunodetected, in accordance with the kinetics of RGC loss quantified with each marker. In addition, there are major morphological differences between microglial cells in ROCs and after ONC, suggesting a different activation pattern between models.

After ONC, Müller cell activation is mostly restricted to their endfeet located at the GCL. In retinal dystrophies, Müller cell hypertrophy is stronger in their somas and stem processes [32,47]. In ROCs, they become activated earlier than after ONC and show signs of activation in their endfeet, somas, and stem processes, mimicking the hypertrophy observed in ischemic insults [36].

Finally, it is important to acknowledge that no model perfectly mimics the disease it is intended to represent, and therefore its shortcomings must be recognized while focusing on the aspects that are relevant.

## 4. Materials and Methods

### 4.1. Animals

Adult C57BL/6 pigmented male mice were obtained from the University of Murcia breeding colony. All animals were treated in accordance with the ARRIVE guidelines, European Union guidelines for the Care and Use of Animals for Scientific Purposes (Directive 2010/63/EU) and the guidelines of the Association for Research in Vision and Ophthalmology (ARVO) Statement for the Use of Animals in Ophthalmic and Vision Research. All procedures were approved by the Ethics and Animal Studies Committee of the University of Murcia, Spain (A1320140704 approved in July 2014, extended on 30 July 2020, valid until 2025).

ONC and RGC retrograde tracing were performed under general anesthesia administered intraperitoneally with a mixture of ketamine (60 mg/kg, Ketolar, Parke-Davies, SL, Barcelona, Spain) and xylazine (10 mg/kg, Rompun, Bayer SA, Barcelona, Spain). 

Analgesia was provided by subcutaneous administration of buprenorphine (0.1 mg/kg; Buprex, Buprenorphine 0.3 mg/mL; Schering-Plough, Madrid, Spain). During and after anesthesia, the eyes were covered with an ointment (Tobrex; Alcon SA, Barcelona, Spain) to prevent corneal desiccation. The animals used were killed by intraperitoneal injection of an overdose of sodium pentobarbital (Dolethal, Vetoquinol; Especialidades Veterinarias, SA, Alcobendas, Madrid, Spain) or by a CO2 exposure (ROCs). 

### 4.2. Experimental Design

To compare RGC degeneration and glial activation in ONC model and in vitro, retinas were analyzed at increasing post injury time points or days in culture (Figure 1A, *n* = 4–6 retinas/ time point/analysis). Intact retinas processed as ROCs or as standard flat-mounts were used as a control (*n* = 4–6 retinas/analysis). To assess microglial phagocytic capacity, retinas were retrogradely traced and analyzed 7 days after optic nerve axotomy or in vitro (Figure 1B).

### 4.3. Retinal Organotypic Cultures (ROCs)

All ROCs were carried out in a laminar flow cabinet under sterile conditions at the Tissue Culture Service (Scientific and Technical Research Area, ACTI) of the University of Murcia/IMIB Arrixaca.

ROCs were performed as previously reported with some modifications [21,22,23]. Briefly, freshly enucleated eyes were quickly (2–4 s) rinsed in EtOH 80% and transferred to DMEM/F-12 (supplemented with 200 mM L-glutamine, 100 units/mL penicillin and 100 µL/mL streptomycin). The cornea and lens were removed, and the retina peeled away from the sclera. After cutting the optic nerve, the retina was placed photoreceptor’s side down on the culture insert (Cell Culture Insert, PIHP01250; Millipore, Darmstadt, Germany). Inserts were placed in the 24 multiwell plate and maintained at 37 °C with 5% CO2 in DMEM/F-12 with 25% HBSS and 25% FBS. Medium was changed two hours after placing the retina on the insert, and thereafter daily. 

### 4.4. Optic Nerve Crush (ONC)

The left optic nerve was accessed intra-orbitally and crushed at 0.5 mm from the optic disk following the methods described previously [8,10,48]. Briefly, an incision was made in the left superior orbital rim and the superoexternal orbital contents were dissected. 

Then, the superior and lateral rectus muscles were removed, and the left optic nerve exposed and crushed thoroughly for 10 s (ONC) at 0.5 mm from the optic disk. After surgery, fundus blood flow was assessed to confirm the absence of retinal ischemia. 

### 4.5. RGC Retrograde Tracing

The retrogradely transported tracer hydroxystilbamidine methanesulfonate (OHSt, Molecular Probes, Leiden, The Netherlands) was diluted at 10% in 0.9% NaCl-10% DMSO and applied to both superior colliculi (SCi) 1 week before the axotomy or euthanasia as previously described [2]. Briefly, after exposing the midbrain, a small pledget of gelatin sponge (Spongostan Film, Ferrosan A/S, Denmark) soaked in the tracer solution was applied over the entire surface of both SCi. The craniotomy was covered with Spongostan and the skin sutured with 4/0 silk (Lorca Marín, Murcia, Spain).

### 4.6. Tissue Preparation

After ONC, animals were perfused transcardially with 0.9% saline followed by 4% paraformaldehyde (PFA) in 0.1 M phosphate buffer, and the eyes were fixed for a further hour in 4% PFA. Thereafter, the retinas were dissected as flattened whole mounts [2,3,48]. For ROCs, inserts with the explants still attached to them were immersed in 4% PFA overnight at 4 °C. 

### 4.7. Immunohistofluorescence 

#### 4.7.1. Whole Mount Retinas

RGCs and glial cells were immunodetected in whole mounts as reported [12,30,48]. Briefly, flat-mount retinas were permeabilized by freezing them for 15 min at −80 °C in PBS-0.5% Triton^®^ X-100. Next, they were incubated overnight at 4 °C with the primary antibodies (Table 1) diluted in blocking buffer (PBS-2%Triton^®^ X-100 with 2% normal donkey and/or goat serum, NDS/NGS). Secondary detection was carried out for 2 h at room temperature with Alexa–Fluor conjugated secondary antibodies (1:500 in PBS-2%Triton^®^ X-100, Molecular Probes, Thermo Fisher, Madrid, Spain).

#### 4.7.2. Retinal Organotypic Cultures

ROCs were permeabilized and blocked for 90 min at room temperature in PBS-0.2% Triton^®^ X-100 with 5% NDS and/or NGS. Then, they were incubated at 4 °C for 24 h with the primary antibodies diluted in the same solution. 

Next, explants were washed 6 × 10 min in 0.1% Triton^®^ X-100 in PBS and then incubated for 2 h with the secondary antibodies diluted in 0.1% Triton^®^ X-100 in PBS. 

Finally, flat-mounts and ROCs were washed thoroughly in PBS, mounted vitreous side up, and covered with mounting solution (Vectashield, Vector Laboratories, Palex Medical, Barcelona, Spain).

### 4.8. Image Acquisition, Quantification, and Analysis

Samples were examined and photographed using an epifluorescence microscope (Leica DM6B, Leica Microsytems, Wetzlar, Germany) or by confocal microscopy (Leica SP8) at the Microscopy University Service of the University of Murcia/IMIB Arrixaca.

Four images from the central-medial retina and four from the periphery (at ~0.5 and 1mm from the optic nerve, respectively) were taken from each sample with a 20x objective. Immunodetected cells were manually dotted in Adobe Photoshop^®^ CS 8.0.1 and afterwards the dots automatically quantified using Image-Pro^®^ Plus program.

### 4.9. Statistics

Data are presented as mean ± standard deviation (S.D.) and analyzed with GraphPad Prism v.8 (GraphPad San Diego, CA, USA) using nonparametric Kruskal–Wallis test. Differences were considered significant when *p* < 0.05. X, Y regression analyses were performed using as variables time after lesion/in vitro (X, independent variable) and cell density (Y, dependent variable) for each model and marker.

## 5. Conclusions

The course of RGC death in ROCs and after ONC is similar up to 5 days, meaning that, during this therapeutic window, ROCs may be useful to study new RGC protective drugs. Long-term experiments, however, should be carried out in vivo.

However, ROCs are not a very precise model for testing specific neuroprotective therapies for axotomized RGCs. After ONC only RGCs are injured, while in ROCs there are other factors that may influence therapies aimed to rescue axotomized RGCs. For instance, ROCs cause the concomitant death of RGCs and photoreceptors and a glial reactivity that differs of that observed after ONC alone. Nevertheless, because in ROCs both RGCs and photoreceptors die, they are a very good model to study the interplay between both degenerations such as those observed in in vivo models of ischemia-reperfusion [49]. 

On the other hand, ROCs have a more controlled environment for the use of drugs or adenovirus treatments. Finally, it is important to have in mind that, in ROCs and regardless of the neuronal population under study, the systemic contribution is lost, which may alter the efficacy of the same therapy when tested in vivo.

## Figures and Tables

**Table 1:** Primary antibodies used.

Antigen	Expressed in	Host	Dilution	Company	Cat. Number
Brn3a	RGCs	Mouse	1:300	Millipore	MAB1585
RBPMS	RGCs	Rabbit	1:750	Genetex	GTX118619
β-III Tubulin	RGCs	Rabbit	1:1000	Santa Cruz Biotechnologies	Sc-5274
γ-synuclein	RGCs	Mouse	1:1000	Abnova	H00006623-M01
Iba1	Microglial cells	Rabbit	1:500	Abcam	Ab178846
GFAP	Astrocytes and activated Müller cells	Rabbit	1:500	Merck	G9269
		Goat	1:500	Abcam	Ab53554
Vimentin	Müller cells	Goat	1:250	Santa Cruz Biotechnologies	Sc-7557

## Data Availability

All data generated or analyzed during this study are included in this published article, or if absent are available from the corresponding author upon reasonable request.
